# Nitrosative stress inhibits aminoacylation and editing activities of mitochondrial threonyl-tRNA synthetase by S-nitrosation

**DOI:** 10.1093/nar/gkaa471

**Published:** 2020-06-02

**Authors:** Wen-Qiang Zheng, Yuying Zhang, Qin Yao, Yuzhe Chen, Xinhua Qiao, En-Duo Wang, Chang Chen, Xiao-Long Zhou

**Affiliations:** State Key Laboratory of Molecular Biology, Shanghai Institute of Biochemistry and Cell Biology, Center for Excellence in Molecular Cell Science, Chinese Academy of Sciences, Shanghai 200031, China; School of Life Science and Technology, ShanghaiTech University, Shanghai 201210, China; University of Chinese Academy of Sciences, Beijing 100049, China; National Laboratory of Biomacromolecules, CAS Center for Excellence in Biomacromolecules, Institute of Biophysics, Chinese Academy of Sciences, Beijing 100101, China; National Laboratory of Biomacromolecules, CAS Center for Excellence in Biomacromolecules, Institute of Biophysics, Chinese Academy of Sciences, Beijing 100101, China; National Laboratory of Biomacromolecules, CAS Center for Excellence in Biomacromolecules, Institute of Biophysics, Chinese Academy of Sciences, Beijing 100101, China; National Laboratory of Biomacromolecules, CAS Center for Excellence in Biomacromolecules, Institute of Biophysics, Chinese Academy of Sciences, Beijing 100101, China; State Key Laboratory of Molecular Biology, Shanghai Institute of Biochemistry and Cell Biology, Center for Excellence in Molecular Cell Science, Chinese Academy of Sciences, Shanghai 200031, China; School of Life Science and Technology, ShanghaiTech University, Shanghai 201210, China; University of Chinese Academy of Sciences, Beijing 100049, China; University of Chinese Academy of Sciences, Beijing 100049, China; National Laboratory of Biomacromolecules, CAS Center for Excellence in Biomacromolecules, Institute of Biophysics, Chinese Academy of Sciences, Beijing 100101, China; Beijing Institute for Brain Disorders, Capital Medical University, Beijing 100069, China; State Key Laboratory of Molecular Biology, Shanghai Institute of Biochemistry and Cell Biology, Center for Excellence in Molecular Cell Science, Chinese Academy of Sciences, Shanghai 200031, China; University of Chinese Academy of Sciences, Beijing 100049, China

## Abstract

Structure and/or function of proteins are frequently affected by oxidative/nitrosative stress via posttranslational modifications. Aminoacyl-tRNA synthetases (aaRSs) constitute a class of ubiquitously expressed enzymes that control cellular protein homeostasis. Here, we found the activity of human mitochondrial (mt) threonyl-tRNA synthetase (hmtThrRS) is resistant to oxidative stress (H_2_O_2_) but profoundly sensitive to nitrosative stress (S-nitrosoglutathione, GSNO). Further study showed four Cys residues in hmtThrRS were modified by S-nitrosation upon GSNO treatment, and one residue was one of synthetic active sites. We analyzed the effect of modification at individual Cys residue on aminoacylation and editing activities of hmtThrRS *in vitro* and found that both activities were decreased. We further confirmed that S-nitrosation of mtThrRS could be readily detected *in vivo* in both human cells and various mouse tissues, and we systematically identified dozens of S-nitrosation-modified sites in most aaRSs, thus establishing both mitochondrial and cytoplasmic aaRS species with S-nitrosation *ex vivo* and *in vivo*, respectively. Interestingly, a decrease in the S-nitrosation modification level of mtThrRS was observed in a Huntington disease mouse model. Overall, our results establish, for the first time, a comprehensive S-nitrosation-modified aaRS network and a previously unknown mechanism on the basis of the inhibitory effect of S-nitrosation on hmtThrRS.

## INTRODUCTION

Protein biosynthesis is an essential and fundamental cellular activity in three domains of life, and it includes several sequential steps. The first step of protein biosynthesis involves charging tRNAs with their cognate amino acids by a group of housekeeping enzymes, aminoacyl-tRNA synthetases (aaRSs) (1,2). This reaction, aminoacylation, usually proceeds in two steps, including the activation of amino acids (with the production of the intermediate aminoacyl-AMP) and the subsequent transfer of the amino acid moiety of aminoacyl-AMP to the A76 of a tRNA to form the material of protein biosynthesis, aminoacyl-tRNA (1,2). Some aaRSs are prone to misactivate structurally similar noncognate amino acids and then generate mischarged aminoacyl-tRNAs, which are then excluded by a proofreading (editing) reaction to ensure translational fidelity (3,4). For example, in addition to those from *Mycoplasma* species, nearly all threonyl-tRNA synthetases (ThrRSs) from bacteria, archaea, eukaryotic cytoplasm and mitochondria misactivate noncognate Ser, and the resultant Ser-AMP and/or Ser-tRNA^Thr^ are removed by editing at the pretransfer and/or posttransfer editing levels (5–12).

There are two separate translational machineries in human beings, the cytoplasmic and mitochondrial machineries (13,14). AaRSs and tRNAs in the cytoplasmic translation machinery in mammalian cells are encoded by nuclear genes in the cells. The mitochondrial genome encodes 22 tRNAs, except two genes of tRNA^Ser^ and tRNA^Leu^, only one gene encodes each of the other 18 tRNAs; however, all mitochondrial aaRSs are encoded by the nuclear genome and transported into mitochondria after synthesis in the cytoplasm (13,15,16). The human mitochondrial genome encodes 13 proteins, which are all essential members of the oxidative phosphorylation (OXPHOS) complex; therefore, mitochondrial protein biosynthesis is a critical process controlling a series of key cellular activities, the functional disturbance of which leads to various mitochondrial diseases (17). For instance, human mitochondrial (mt) ThrRS (hmtThrRS), with aminoacylation and editing activities, is encoded by nuclear *TARS2*, and its active sites are well conserved among ThrRSs in bacteria, eukaryotic cytoplasm and mitochondria (12,18). A pathogenic point mutation in hmtThrRS leads to both structural and functional defects and causes a severe mitochondrial disorder due to impaired mitochondrial protein synthesis and function (12,19).

During various cellular activities, such as OXPHOS in mitochondria and under other physiological or pathological stresses, cells generate reactive oxygen species (ROS) or reactive nitrogen species (RNS), which have pleiotropic effects on cellular targets that lead to various downstream results, such as protein posttranslational modifications (PTMs) (20,21). A growing body of evidence has reported that low concentrations of ROS/RNS regulate redox signaling through reversible redox modification of key redox-sensitive residues (usually cysteine (Cys) residues) in proteins serving as redox sensors (22). The various redox PTMs include S-sulfenylation (-SOH), S-sulfinylation (-SO_2_H), S-sulfonylation (-SO_3_H), S-nitrosation (-SNO), S-sulfhydration (-SSH), S-glutathionylation (-SSG), and disulfide bond formation (23). S-nitrosation, the covalent attachment of an NO group to the thiol side chain of Cys (Supplementary Figure S1), is emerging as a critical modification involved in the regulation of many physiological or pathological processes (24–26). The reported physiological concentration of the nitric oxide *in vivo* varies among different reports, ranging from 100 pM to 1 μM, depending on various cells, tissues or treatments (27). Our previous study showed that S-nitrosation modification regulate the activity (28), localization (29), stability and protein-protein interaction of target proteins (30).

Redox modification of components in protein biosynthesis, including aaRSs and tRNA modification enzymes, were previously discovered by targeting Cys residues. A Cys residue at the active site in the editing domain of *Escherichia coli* ThrRS (*Ec*ThrRS) is modified upon H_2_O_2_ treatment *in vitro* to Cys sulfenic acid, leading to compromised editing function and mistranslation of Thr codons as Ser due to the synthesis of mischarged Ser-tRNA^Thr^*in vivo* (31,32). Similarly, it has been recently reported that oxidative stress leads to oxidation of *Salmonella enterica* phenylalanyl-tRNA synthetase (*Se*PheRS) and a conformational change to generate a partially unstructured protein. This alteration in the structure increases the extent of the editing of mischarged Tyr-tRNA^Phe^ but has little effect on its aminoacylation activity (33). Exposure of mammalian cells to oxidative chemicals, such as H_2_O_2_ or arsenite, stimulates an approximate 10-fold increase in the mis-aminoacylation of non-tRNA^Met^s with Met, which is likely mediated by the phosphorylation of human methionyl-tRNA synthetase (MetRS). The resultant Met-mischarged tRNAs are readily used in protein biosynthesis, and Met misincorporation into the proteome is suggested to be beneficial for preventing further oxidation at critical sites during the cell response to oxidative stress (34). Similar observations have also been described in bacteria and yeasts (35). Indeed, both ThrRS and MetRS have been found to be sulfenic acid-modified in HeLa cells (36). In addition to the modulation of tRNA aminoacylation by the modification of aaRSs, other processes, including tRNA modification, are regulated by ROS. For example, high-throughput modification analyses have revealed that the levels of several tRNA modifications (including m^5^C, Cm, m_2_^2^G and t^6^A) are altered upon H_2_O_2_ exposure, possibly due, in part, to the modification of tRNA modification enzymes (37).

Compared with H_2_O_2_, a representative reagent of ROS, the effect of RNS on aaRSs has not been reported. In the current study, we used S-nitrosoglutathione (GSNO), a major physiological NO derivative *in vivo*, as an RNS donor reagent to explore the regulation of aaRSs by S-nitrosation modification.

## MATERIALS AND METHODS

### Reagents

L-Thr, L-Ser, NTP (CTP, ATP, UTP and GTP), GMP, Tris-base, MgCl_2_, NaCl, dithiothreitol (DTT), and tetrasodium pyrophosphate were purchased from Sigma (St. Louis, MO, USA). Pyrophosphatase (PPiase) was obtained from Roche Life Science (Shanghai, China). [^32^P]tetrasodium pyrophosphate (NEX019001MC) and [α-^32^P]ATP (BLU503H250UC) were purchased from PerkinElmer (Shelton, CT, USA). KOD-Plus mutagenesis kits were obtained from TOYOBO (Osaka, Japan). All restriction endonucleases, T4 DNA ligase, and T4 polynucleotide kinase were obtained from Thermo Scientific (Waltham, MA, USA). Ni^2+^-NTA Superflow resin was purchased from Qiagen Inc. (Hilden, Germany). DNA sequencing and primer synthesis were performed by Biosune (Shanghai, China). GSNO was synthesized from glutathione with acidified nitrite as described previously (38). Briefly, GSH reacted with the equimolar sodium nitrite at 4°C in HCl (625 mM) for 45 min in the dark. Then the 2.5 volumes of acetone were added and the mixture was stirred constantly for 20 min. GSNO was washed by acetone and dried under vacuum. Prepared GSNO was quantified by the absorbance of 334 nm.

### Gene cloning, mutagenesis, and expression and protein purification

The plasmids expressing the mature form of hmtThrRS (pET28-hmtThrRS) and mouse cytoplasmic ThrRS (pET28-mThrRS) were constructed previously (11,12). Gene mutagenesis was performed according to the procedures provided with the KOD-Plus mutagenesis kit. All constructs were confirmed by DNA sequencing by Biosune (Shanghai, China). *Escherichia coli* BL21(DE3) was transformed with various constructs. Gene overexpression was induced at the mid-log phage (*A*_600_ = 0.6) with 200 μM isopropyl β-d-1-thiogalactopyranoside (IPTG) at 20°C overnight. Protein purification was performed according to a previously described method (12). No reducing reagents, including DTT or 2-mercaptoethanol, were used in the purification procedures.

### tRNA preparation and ^32^P-labeling

Both hmtThrRS and mThrRS aminoacylate purified fully modified *E. coli* tRNA^Thr^(UGU) and human mitochondrial tRNA^Thr^ transcript with similar efficiency (11); therefore, *E. coli* tRNA^Thr^(UGU) (tRNA^Thr^) was used in this study. The construct for expressing tRNA^Thr^ was previously constructed (10) and was introduced into the *E. coli* MT102 strain. tRNA gene expression and purification were performed with previously established procedures (10). The obtained tRNA^Thr^ was of high purity and quality, as determined by UREA-PAGE analysis and Thr-accepting measurement. ^32^P-labeling of the 3′-end of the tRNA^Thr^ was performed at 37°C in a mixture containing 60 mM Tris–HCl (pH 8.0), 12 mM MgCl_2_, 20 μM tRNA^Thr^, 0.5 mM DTT, 20 μM ATP, 50 μM tetrasodium pyrophosphate, 0.666 μM [α-^32^P]ATP and 10 μM *E. coli* CCA-adding enzyme (CCase) for 5 min. Finally, 0.8 U/μl PPiase was added to the mixture for 2 min. [^32^P]tRNA^Thr^ was extracted with phenol/chloroform twice, precipitated with ethanol overnight at −20°C and dissolved in 5 mM MgCl_2_.

### *In vitro* activities determination

GSNO was freshly prepared immediately prior to activity determination and kept in the dark. The concentration of 1 μM mThrRS or hmtThrRS was initially incubated in the absence or presence of 1 mM GSNO at 37°C for 1 h. The GSNO untreated or treated mThrRS or hmtThrRS samples were used as a 10-fold stock in the activity determination assay (the final concentration of enzyme and GSNO in reactions was 100 nM and 100 μM, respectively), or as a 3.33-fold stock in the activity determination of C409 and 4S mutants due to low activity (the final concentration of enzyme and GSNO in reactions was 300 nM and 300 μM, respectively).

The rate of amino acid activation by hmtThrRS was assayed by an ATP-PPi exchange measurement, which was carried out at 37°C in a reaction mixture containing 60 mM Tris-HCl (pH 7.5), 10 mM MgCl_2_, 2.5 mM ATP, 2 mM tetrasodium [^32^P]pyrophosphate and 1 mM Thr with final concentration of 100 nM hmtThrRS untreated or treated with GSNO. At various time intervals, 15-μl aliquots of the reaction mixture was quenched with a solution containing 2% activated charcoal, 3.5% HClO_4_ and 50 mM tetrasodium pyrophosphate to a final volume of 200 μl. The solution was then filtered through a Whatman GF/C filter and washed with 20 ml of 10 mM tetrasodium pyrophosphate solution and 10 ml of 100% ethanol. The filters were dried, and the [^32^P]ATP was measured using a scintillation counter (Beckman Coulter).

To determine the effect of GSNO on the aminoacylation of mThrRS and hmtThrRS, aminoacylation was tested in a reaction mixture containing 60 mM Tris–HCl (pH 7.5), 10 mM MgCl_2_, 1 mM Thr, 10 μM unlabeled tRNA^Thr^ and ∼40000 cpm [^32^P]tRNA^Thr^ with final concentration of 100 nM mThrRS or hmtThrRS untreated or treated with GSNO. Final concentrations of 300 nM C409 or 4S were used in their aminoacylation determinations. The effect of GSNO on the aminoacylation of *Ec*ThrRS (final concentration of 100 nM) was determined in a similar reaction buffer except at a final concentration of 500 μM GSNO. For the mischarging assays, 100 mM Ser was used instead of 1 mM Thr. The effect of H_2_O_2_ on the aminoacylation of mThrRS and hmtThrRS was determined in similar reactions except that 1 μM mThrRS and hmtThrRS was initially incubated in the absence or presence of 5 mM H_2_O_2_ at 37°C for 30 min and then used as a 10-fold stock.

The mischarged Ser-[^32^P]tRNA^Thr^ was generated by editing-defective hmtThrRS-H133A/H137A mutant (12) in a mischarging reaction containing 1000 mM Ser. The posttransfer editing was determined in a reaction solution containing 60 mM Tris–HCl (pH 7.5), 10 mM MgCl_2_, 2 μM Ser-[^32^P]tRNA^Thr^ and final concentrations of 375 nM hmtThrRS or its variants untreated or treated with GSNO. For the aminoacylation, mischarging and posttransfer editing assays, at specific time points, samples were taken for ethanol precipitation with NaAc (pH 5.2) at −20°C overnight. The precipitated samples were centrifuged (10 000 *g*) at 4°C for 30 min, dried at room temperature for 30 min and digested with 6 μl of nuclease S1 (25 U) for 2 h at 37°C. Upon treatment with nuclease S1, aminoacyl-[^32^P]AMP and [^32^P]AMP is produced from aminoacyl-[^32^P]tRNA and free [^32^P]tRNA, respectively. Samples (2 μl) of the digestion mixture were loaded and separated by thin layer chromatography (TLC) in 0.1 M NH_4_Ac and 5% acetic acid. The plates were visualized by phosphorimaging, and the data were analyzed using Multi-Gauge Version 3.0 software (Fujifilm). The amount of aminoacyl-[^32^P]AMP produced was calculated by multiplying the total amount of tRNA^Thr^ by the relative level of charged tRNA^Thr^ in the aliquots: [aminoacyl-[^32^P]AMP/(aminoacyl-[^32^P]AMP + [^32^P]AMP)].

### Cys residue titration with DTNB

The number of thiol groups in the purified hmtThrRS was determined with DTNB. A total of 2.7 μM protein was incubated with 1 mM DTNB in reaction buffer (0.1 M sodium phosphate, pH 8.0, containing 1 mM EDTA) at 25°C for 5 min. Absorbance was measured at 412 nm, and the sulfhydryl concentrations were calculated using a molar absorption coefficient of 14 150 under native conditions and 13 880 in the presence of 6 M guanidine hydrochloride.

### Antibodies

The following antibodies were used: anti-β-Actin (KM9001) from Sungene Biotech and the preparation of anti-ThrRS and anti-GluProRS has been described in previous reports (11,39). Purified mature hmtThrRS and mitochondrial AlaRS (mtAlaRS) (40,41) were used as antigens to generate anti-mtThrRS or anti-mtAlaRS antibodies, respectively.

### Animals

R6-1 mice were bred in a specific pathogen-free barrier facility of the Institute of Biophysics, Chinese Academy of Science. The Animal Center approved all procedures involving animals.

### Cell cultures and transfection

HEK293T and HeLa cells were cultured in Dulbecco's modified Eagle's medium (HyClone, SH30243.01) supplemented with 10% fetal bovine serum (GIBCO, 10019-141), 100 U/ml penicillin, and 100 mg/ml streptomycin (HyClone, SV30010). The cells were transfected with Lipofectamine 2000 (Life Technologies) according to the manufacturer's instructions.

### Western blot analysis

Protein extracts from cells and tissues were separated by 10% SDS-PAGE. The separated proteins were transferred to a nitrocellulose filter membrane (Millipore). The membrane was treated with 5% (w/v) fat-free milk in TBST for 1 h and incubated with the indicated antibody overnight at 4°C, followed by incubation with peroxidase-conjugated anti-rabbit or mouse IgG (Santa Cruz) for 1 h. The bands were visualized with an ECL western blot detection kit (Pierce).

### Mitochondria isolation

Mitochondria were isolated according to the procedures described by Wieckowski *et al.* (42). Briefly, HEK293T cells were cultured as described above. The cells were harvested and washed with PBS solution at least three times. The cell pellets were then suspended in ice-cold starting buffer (225 mM mannitol, 75 mM sucrose and 30 mM Tris–HCl pH 7.4) and homogenized using an ice-cold Teflon pestle. The integrity of the homogenized cells was determined by examination under a light microscope at specific time intervals. The homogenate was then transferred to a 15-ml polypropylene centrifugation tube and centrifuged twice at 600 *g* for 5 min at 4°C. The supernatant was then collected and centrifuged at 7,000 *g* for 10 min at 4°C. Then, the supernatant (cytosolic fraction) was discarded, and the mitochondria were enriched in the pellet.

### Irreversible biotin switch assay procedure (IBP)

The IBP for detecting the S-nitrosation modification was carried out as previously described (43), which is a modified method based on the original biotin switch assay (44). This improved method enables us to eliminate the potential interference of intermolecular disulfide bond in the detection of protein S-nitrosation. Briefly, adult mouse cortex tissues or HeLa cells were lysed in HEN buffer (250 mM HEPES pH 7.7, 1 mM EDTA, 0.1 mM neocuproine) with 1% NP40 (Nonidet *P*-40) and protease inhibitor cocktail. The supernatant was incubated with 2.5% SDS and 20 mM methyl methanethiosulfonate (MMTS) at 50°C for 30 min to block free thiols with frequent vortex. Excess MMTS was removed by 80% ice-cold acetone precipitation followed by centrifugation at 2,000 *g* for 10 min. The precipitation was repeated three times. Nitrosothiols in precipitate was reduced and biotinylated in HENS buffer with 10 mM ascorbate and 0.4 mM biotin-maleimide by incubation at room temperature for 2 h. The excess biotin-maleimide was removed by 80% ice-cold acetone precipitation as described above. The protein pellet was resuspended in HENS buffer (HEN buffer with 2.5% SDS) containing 200 mM DTT and incubated at 100°C for 10 min to eliminate potential intermolecular disulfide bonds. The biotinylated proteins were purified by streptavidin-agarose (50–100 μl/sample) after addition two volumes of neutralization buffer (250 mM HEPES, pH 7.7, 100 mM NaCl, 1 mM EDTA, 0.1 mM neocuproine) at 4°C overnight. The agarose was washed three times by neutralization buffer. Finally, the proteins were eluted by HENS buffer at 100°C for 15 min. The eluted enriched proteins were analyzed by immunoblotting with the relevant antibodies.

### S-nitrosation proteomic analysis

Purified protein pretreated with GSNO (500 μM, 1 h) prepared according to the IBP was separated by SDS-PAGE. After electrophoresis, the protein was excised from gels and digested. After the mixed samples were suspended, Spin Tip C18 desalination was performed, and the peptide was analyzed by MS to detect biotin-modified Cys residues.

The iodoTMT labeling procedure was conducted by using iodoacetyl tandem mass tag™ (iodoTMT™) reagents (90103, Thermo Scientific, USA) according to the manufacturer's instructions. Briefly, purified human mitochondria pretreated with GSNO (1 mM, 1 h) or separated mouse cortexes were lysed in HENS buffer, the free Cys residues were blocked with MMTS, and the S-nitrosated Cys residues were labeled with iodoTMT reagent and enriched with anti-TMT antibody. Peptides were finally eluted and resuspended in 5% acetonitrile/0.1% formic acid. The multiplexed quantitative MS data were collected by using a Q Exactive mass spectrometer equipped with an easy n-LC 1000 HPLC system (Thermo Scientific) in data-dependent acquisition mode. The raw data obtained from Q Exactive were analyzed with Proteome Discovery version 1.4 using the Sequest HT search engine for protein identification and Percolator for a false discovery rate analysis. The UniProt mouse protein database was used to search data from the mouse samples. Protein quantification was also performed with Proteome Discovery version 1.4 using the ratio of reporter ion intensity obtained from the MS/MS spectra. Only unique peptides of proteins or protein groups were selected for relative protein quantification. Normalization to the median value of the protein in each sample was used to correct experimental bias.

## RESULTS

### Activities of both cytosolic and mitochondrial ThrRSs are not affected by H_2_O_2_

**Figure 1.**
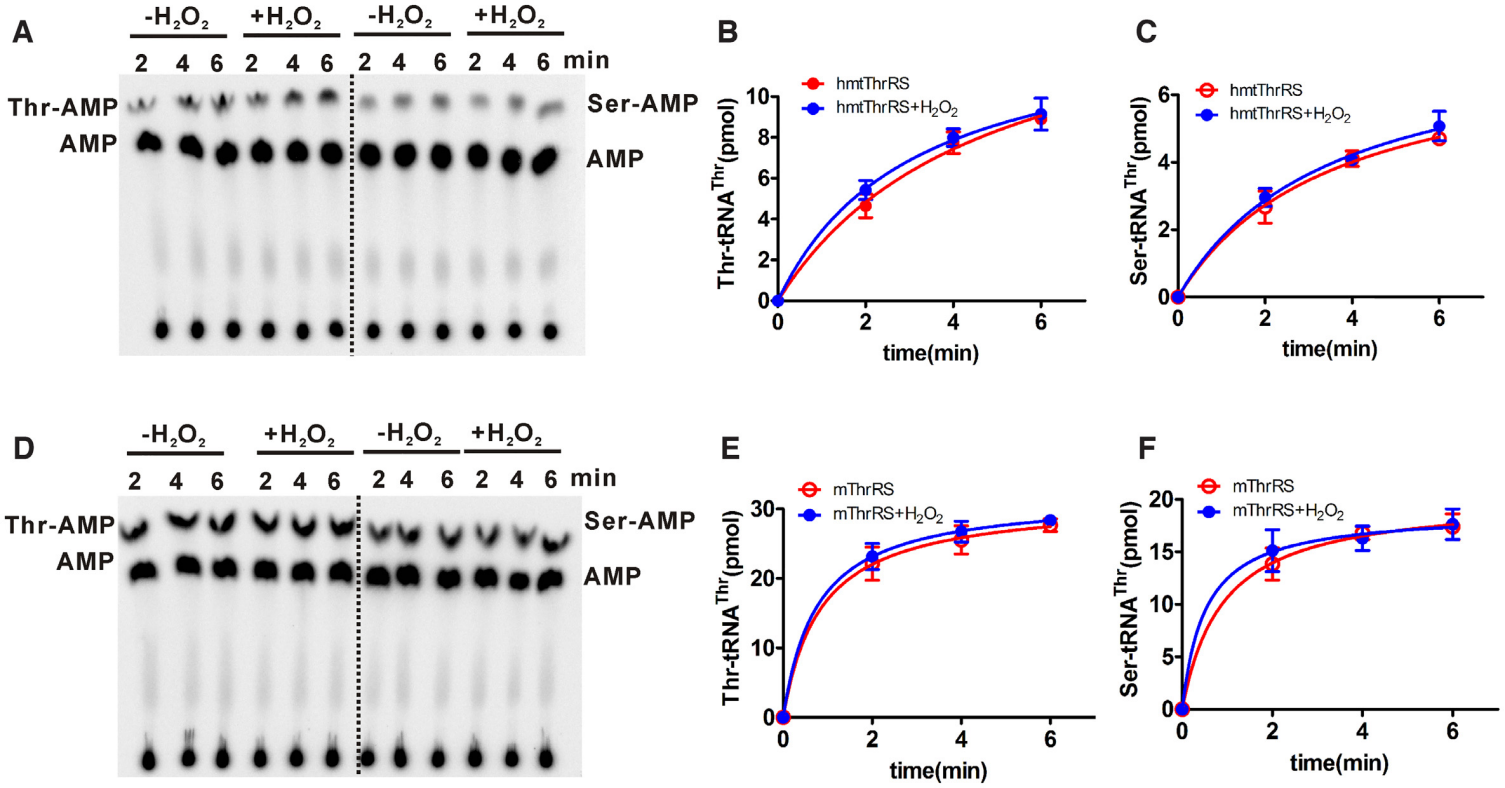
H_2_O_2_ has no effects on the aminoacylation or mischarging of mitochondrial or cytoplasmic ThrRSs. (**A**) A representative graph showing aminoacylation (left) or mischarging (right) of [^32^P]tRNA^Thr^ with Thr and Ser, respectively, by hmtThrRS, in the absence or presence of H_2_O_2_ treatment as indicated. Free [^32^P]tRNA^Thr^ and (mis)charged [^32^P]tRNA^Thr^ are represented by [^32^P]AMP and Thr/Ser-[^32^P]AMP after digestion by nuclease S1. Amount of Thr-[^32^P]tRNA^Thr^ (**B**) or Ser-[^32^P]tRNA^Thr^ (**C**) catalyzed by hmtThrRS without (red) or with (blue) H_2_O_2_ treatment, as in (A). (**D**) A representative graph showing aminoacylation (left) or mischarging (right) of [^32^P]tRNA^Thr^ with Thr and Ser, respectively, by mThrRS, in the absence or presence of H_2_O_2_ treatment as indicated. Amount of Thr-[^32^P]tRNA^Thr^ (**E**) and Ser-[^32^P]tRNA^Thr^ (**F**) catalyzed by mThrRS without (red) or with (blue) H_2_O_2_ treatment, as in (**D**). Reaction conditions were described in detail in the ‘MATERIALS AND METHODS’.

**Figure 2.**
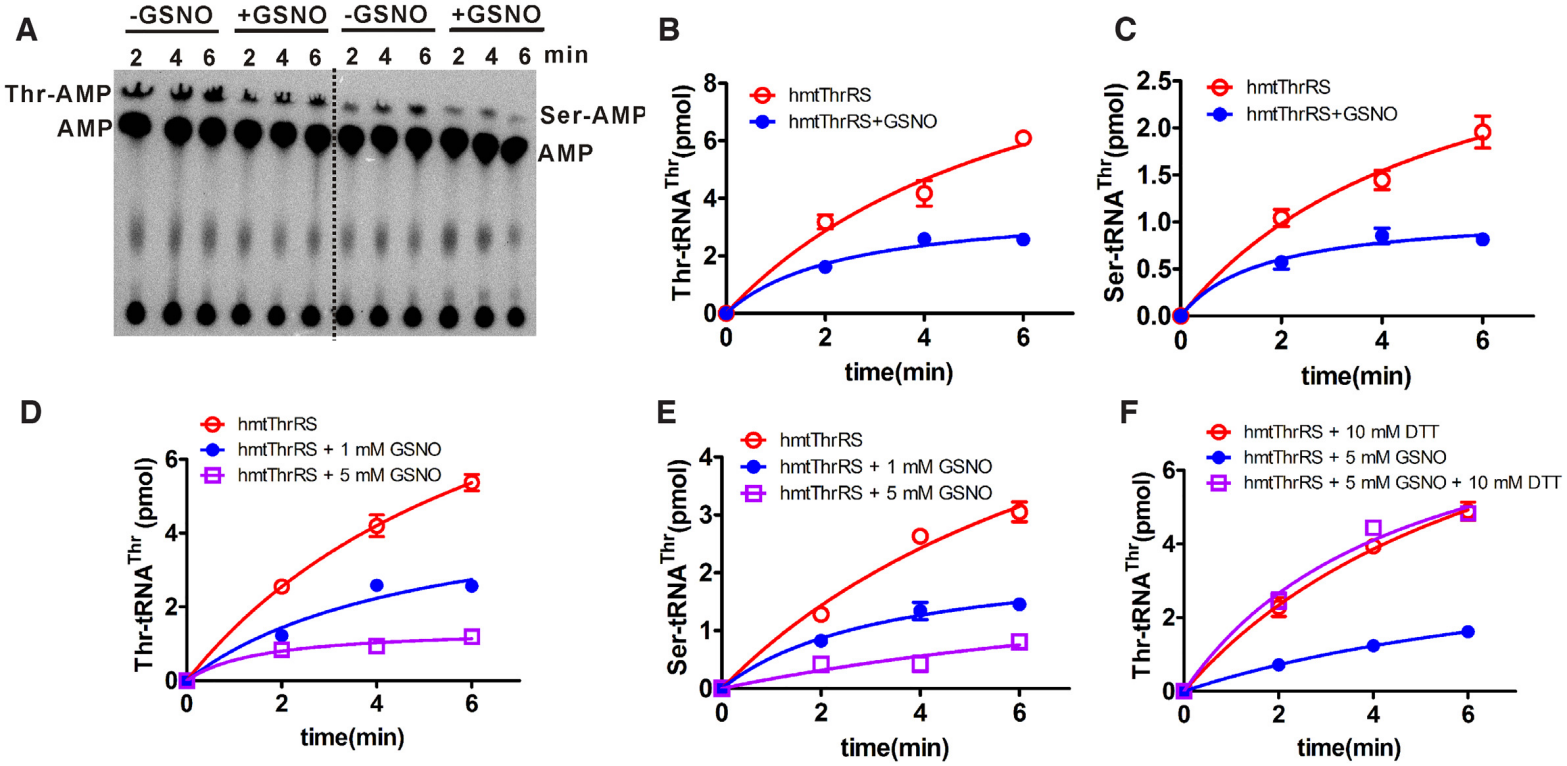
GSNO decreases both the aminoacylation and mischarging activities of hmtThrRS in a dose-dependent manner. (**A**) A representative graph showing aminoacylation (left) and mischarging (right) of [^32^P]tRNA^Thr^ with Thr and Ser, respectively, by hmtThrRS in the absence or presence of GSNO treatment, as indicated. Free [^32^P]tRNA^Thr^ and (mis)charged [^32^P]tRNA^Thr^ are represented by [^32^P]AMP and Thr/Ser-[^32^P]AMP after digestion by nuclease S1. Quantitative analysis of Thr-[^32^P]tRNA^Thr^ (**B**) and Ser-[^32^P]tRNA^Thr^ (**C**) generated by hmtThrRS without (red) or with (blue) GSNO treatment in (A). Aminoacylation (**D**) and mischarging (**E**) activity of hmtThrRS was determined without GSNO incubation or after treatment with increasing concentrations (1 mM (blue) or 5 mM (purple)) of GSNO. (**F**) Inhibition of aminoacylation after 5 mM GSNO treatment (blue) was readily eliminated by 10 mM DTT addition to the reaction mixture (purple). Reaction conditions were described in detail in the ‘MATERIALS AND METHODS’.

Considering the sensitivity of *Ec*ThrRS to H_2_O_2_ (31), we first assayed H_2_O_2_ effects on the aminoacylation activity of hmtThrRS. Aminoacylation of tRNA^Thr^ by hmtThrRS was performed in the absence or presence of H_2_O_2_ treatment. The synthesis of Thr-tRNA^Thr^ was obviously not affected by H_2_O_2_ (Figure 1A, left panel and Figure 1B). Furthermore, mischarging of tRNA^Thr^ with noncognate Ser was determined without or with H_2_O_2_ treatment; a similar level of Ser-tRNA^Thr^ was formed (Figure 2A, right panel and Figure 1C), suggesting that the editing activity of hmtThrRS was also not influenced by H_2_O_2_.

Subsequently, to understand the effect of H_2_O_2_ on the aminoacylation and mischarging activities of cytoplasmic ThrRS, we treated mouse cytoplasmic ThrRS (mThrRS) with H_2_O_2_ and found no effects on either the aminoacylation or mischarging activity of mThrRS (Figure 1D–F). Therefore, these data suggested that the *in vitro* aminoacylation and editing activities of both mammalian cytoplasmic and mitochondrial ThrRSs were not affected by H_2_O_2_.

### GSNO inhibits the aminoacylation and mischarging of tRNA^Thr^ by hmtThrRS

In addition to ROS, RNS is able to affect enzymatic activities (45). To understand whether RNS can potentially regulate hmtThrRS activities, hmtThrRS was preincubated with a representative reagent of RNS, GSNO, and the aminoacylation activity of hmtThrRS before and after GSNO treatment was assayed and compared. The data showed that the synthesis of Thr-tRNA^Thr^ was obviously decreased after GSNO treatment (Figure 2A, left panel and Figure 2B). This inhibitory effect of GSNO was also found in the mischarging assays (Figure 2A, right panel and Figure 2C). We then determined the amino acid activation activity of hmtThrRS in the absence or presence of GSNO treatment by an ATP-PPi exchange reaction, and the results showed that the amino acid activation activity was modestly decreased (Supplementary Figure S2). We then treated hmtThrRS with different concentrations (1 mM and 5 mM) of GSNO. The resulting inhibition of both the aminoacylation and mischarging activities of hmtThrRS was dose-dependent, and only little activities were detected after the 5 mM GSNO treatment (Figure 2D, E). Considering that GSNO induces S-nitrosation of proteins, these results suggested that GSNO likely induced the S-nitrosation of hmtThrRS and subsequently affected its aminoacylation and mischarging activities with a possible similar mechanism in activation of both cognate threonine and noncognate serine.

To confirm S-nitrosation of hmtThrRS after GSNO treatment, after pre-incubation of hmtThrRS with 5 mM GSNO (the concentration at which an obvious inhibition of aminoacylation was observed; Figure 2D), DTT was then added to determine the aminoacylation activity of the enzyme. The results showed that DTT could efficiently restore the activity of hmtThrRS after GSNO treatment (Figure 2F), suggesting a reversal of the S-nitrosation modification.

The aminoacylation kinetics of hmtThrRS without or with GSNO incubation were determined. The data showed that, in the presence of 1 mM GSNO, the *k*_cat_ value of hmtThrRS for tRNA was decreased ∼2-fold (0.027 versus 0.013 s^−1^), while its *K*_m_ value was not obviously altered (0.73 versus 0.69 μM), suggesting that S-nitrosation affected the catalytic rate of hmtThrRS but not its tRNA binding capacity. More inhibition was expected with increasing concentrations of GSNO treatment. We have reported that hmtThrRS is a dimer (16). The results of a gel filtration analysis of hmtThrRS in the absence or presence of GSNO showed that the dimerization of hmtThrRS was not affected (Supplementary Figure S3).

Taken together, these data suggested that GSNO induced the reversible S-nitrosation of hmtThrRS, which decreased the aminoacylation and mischarging of tRNA^Thr^ by hmtThrRS.

### Species specificity of GSNO inhibition of aminoacylation

To address whether GSNO-induced inhibition of aminoacylation via S-nitrosation is specific for hmtThrRS or targets other ThrRSs from other species, after incubation without or with 5 mM GSNO, the aminoacylation activity of *Ec*ThrRS was assayed and compared. Although the aminoacylation activity of hmtThrRS at 5 mM GSNO was nearly abolished (Figure 2D), that of *Ec*ThrRS was not affected at all (Supplementary Figure S4A and S4B). The results showed that the tRNA charging activity of *Ec*ThrRS was insensitive to GSNO treatment, suggesting that the sensitivity of aminoacylation by ThrRS to GSNO is species-specific.

### Identification of four Cys residues modified by S-nitrosation in hmtThrRS

**Figure 3.**
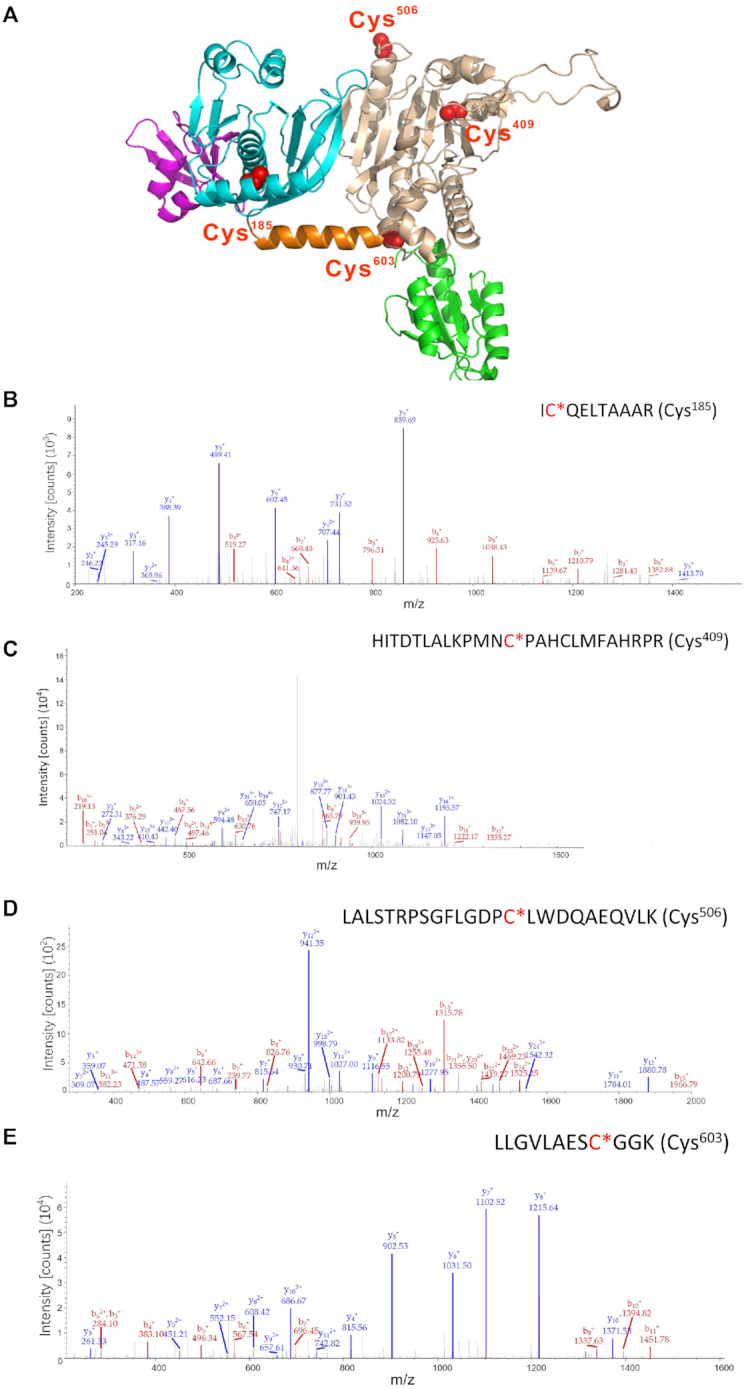
Identification of S-nitrosation-modified Cys residues in hmtThrRS. (**A**) A modeled hmtThrRS structure based on the crystal structure of *Ec*ThrRS (PDB No. 1QF6) with four identified modified Cys residues (Cys^185^, Cy^s409^, Cys^506^ and Cys^603^) indicated by red spheres. Purple, N1 domain; cyan, N2 editing domain; orange, a linker peptide between the editing and aminoacylation domains; wheat, aminoacylation domain; and green, C-terminal tRNA binding domain. (**B–E**) Tandem mass spectrum of the peptides showing S-nitrosation of Cys^185^, Cys^409^, Cys^506^ and Cys^603^ of the GSNO-treated recombinant hmtThrRS protein. Biotin-M-containing peptides are shown, and asterisks indicate the biotinylated Cys residues.

Based on the crystal structure of *E. coli* ThrRS (PDB No. 1QF6) (5), we modeled the structure of hmtThrRS. Mature hmtThrRS contains 699 amino acid residues (Leu^20^-Phe^718^), comprising an N-terminal extension domain (Leu^20^-Thr^60^), N1 domain (Ile^61^-Ser^124^), N2 editing domain (Pro^125^-Pro^282^), linker peptide (Thr^283^-Arg^300^), aminoacylation domain (Asp^301^-Trp^607^), and C-terminal tRNA anticodon binding domain (CTD, Pro^608^-Phe^718^) (12). There are 13 Cys residues, including 4 (Cys^152^, Cys^185^, Cys^233^ and Cys^240^) in the N2 editing domain and 9 (Cys^322^, Cys^409^, Cys^413^, Cys^453^, Cys^463^, Cys^475^, Cys^506^, Cys^554^ and Cys^603^) residues in the aminoacylation domain. Purified hmtThrRS was treated with 5,5′-dithiobis-(2-nitrobenzoic acid) (DTNB), and the Cys number of hmtThrRS was further determined. The data showed that with GndHCl, the unfolded hmtThrRS contained 13.1 Cys residues, which is the theoretical Cys number; without GndHCl, natural hmtThrRS contained 10 Cys residues accessible to DTNB (Supplementary Table S1). After incubation of hmtThrRS with GSNO, an irreversible biotinylation procedure (IBP) combined with mass spectrometry (MS) (IBP-MS method) (Supplementary Figure S5) was performed to identify the potential S-nitrosation-modified Cys residues in hmtThrRS. This method, instead of classical reversible biotinylation, was applied to specifically detect S-nitrosation protein targets without the potential interference of intermolecular disulfide bonds (43). Four Cys residues (Cys^185^, Cys^409^, Cys^506^ and Cys^603^) were identified with S-nitrosation modification with a coverage of 92.34%, which included all the Cys residues (Figure 3A–E) (Supplementary Table S2). By sequence alignment of various ThrRSs, Cys^185^ in the N2 domain of hmtThrRS was found to correspond to Met^124^ in the N2 domain of *Ec*ThrRS and is only conserved in cytoplasmic ThrRSs, ThrRS-like proteins (encoded by *TARS3*) (11,46), and mitochondrial ThrRSs from higher eukaryotes and buried inside the N2 domain (Figure 3A, Supplementary Figure S6). Cys^506^ is a non-conserved residue located at the surface of the aminoacylation domain and points into solution (Figure 3A, Supplementary Figure S6). Cys^603^ is only conserved in mitochondrial ThrRSs, located between aminoacylation and CTD domains and is surface exposed to solution (Figure 3A, Supplementary Figure S6). Among these 4 Cys residues, Cys^409^ is the most relevant because it is absolutely conserved in all ThrRSs and is one of the amino acid activation sites (Figure 3A, Supplementary Figure S6). It corresponds to Cys^334^ in *Ec*ThrRS and constitutes a zinc-binding pocket with His^385^ (His^460^ in hmtThrRS) and His^511^ (His^585^ in hmtThrRS), which is essential for binding Thr and distinguishing Thr from noncognate Val. The mutation of Cys^334^ to Ser has been shown to be lethal for *E. coli in vivo* (47).

### Effect of S-nitrosation at each residue on the aminoacylation and editing activities of hmtThrRS

To understand the role of S-nitrosation at individual Cys residues among the four abovementioned modified Cys residues, four mutants with triple mutations of Cys to Ser were constructed, leaving only one Cys residue as an S-nitrosation candidate: C185 mutant (only Cys^185^ was not mutated to Ser), C409 mutant (only Cys^409^ was not mutated to Ser), C506 mutant (only Cys^506^ was not mutated to Ser), and C603 mutant (only Cys^603^ was not mutated to Ser). In addition, another mutant, with quadruple mutations from Cys to Ser (4S), was also constructed.

**Figure 4.**
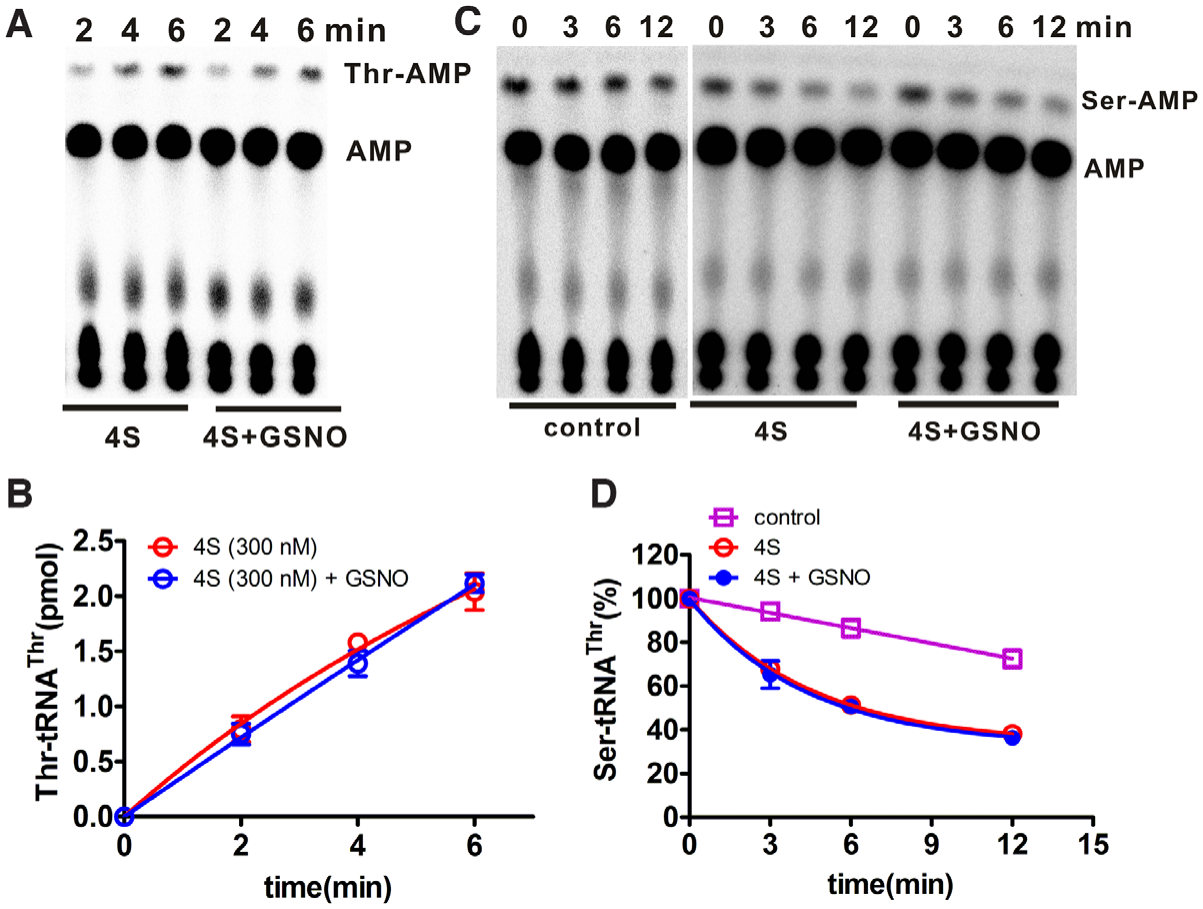
Elimination of the GSNO-induced inhibition of aminoacylation and editing. A representative graph showing aminoacylation of [^32^P]tRNA^Thr^ (**A**) or posttransfer editing of pre-formed Ser-[^32^P]tRNA^Thr^ (**C**) by the 4S mutant in the absence or presence of GSNO treatment, as indicated. 300 nM 4S mutant was used for aminoacylation determination due to its low activity. Results from the quantitative analysis of Thr-[^32^P]tRNA^Thr^ (**B**) and hydrolysis of Ser-[^32^P]tRNA^Thr^ (**D**) as catalyzed by the 4S mutant without (red) or with (blue) GSNO treatment in (A) and (C), respectively. A control reaction (purple) represents the spontaneous hydrolysis of Ser-[^32^P]tRNA^Thr^ without the addition of enzyme in (D). Reaction conditions were described in detail in the ‘Materials and Methods’.

We measured both the aminoacylation and posttransfer editing activities of four triple mutants without or with 1 mM GSNO treatment. Both activities for the four mutants were obviously inhibited after GSNO incubation compared with those of the untreated enzymes, suggesting that S-nitrosation at each site was able to modify the enzymatic activities (Supplementary Figure S7A–E). In line with the crucial role of Cys^409^ in aminoacylation, the inhibitory effect of GSNO on the C409 mutant was the most prominent among all the triple mutants (Supplementary Figure S7C, left panel). Interestingly, the mutants with mutations of Cys^409^ into Ser (C185, C506, and C603 mutants) maintained obviously decreased, but detectable, aminoacylation activity, suggesting that the mutation of Cys^409^ did not completely abolish aminoacylation (Supplementary Figure S7B, D and E). Importantly, GSNO-induced inhibition of both aminoacylation (Figure 4A, B) and editing (Figure 4C, D) activities was completely eliminated with the 4S mutant, suggesting a lack of functional S-nitrosation after the 4 Cys residues were blocked.

These data suggested that S-nitrosation of hmtThrRS was not random and that modification at each site had an inhibitory effect on both the aminoacylation and editing activities of the enzyme, which was relieved by mutations of all four Cys residues.

### S-nitrosation of mitochondrial ThrRS is readily detected in human cell lines and mouse tissues

**Figure 5.**
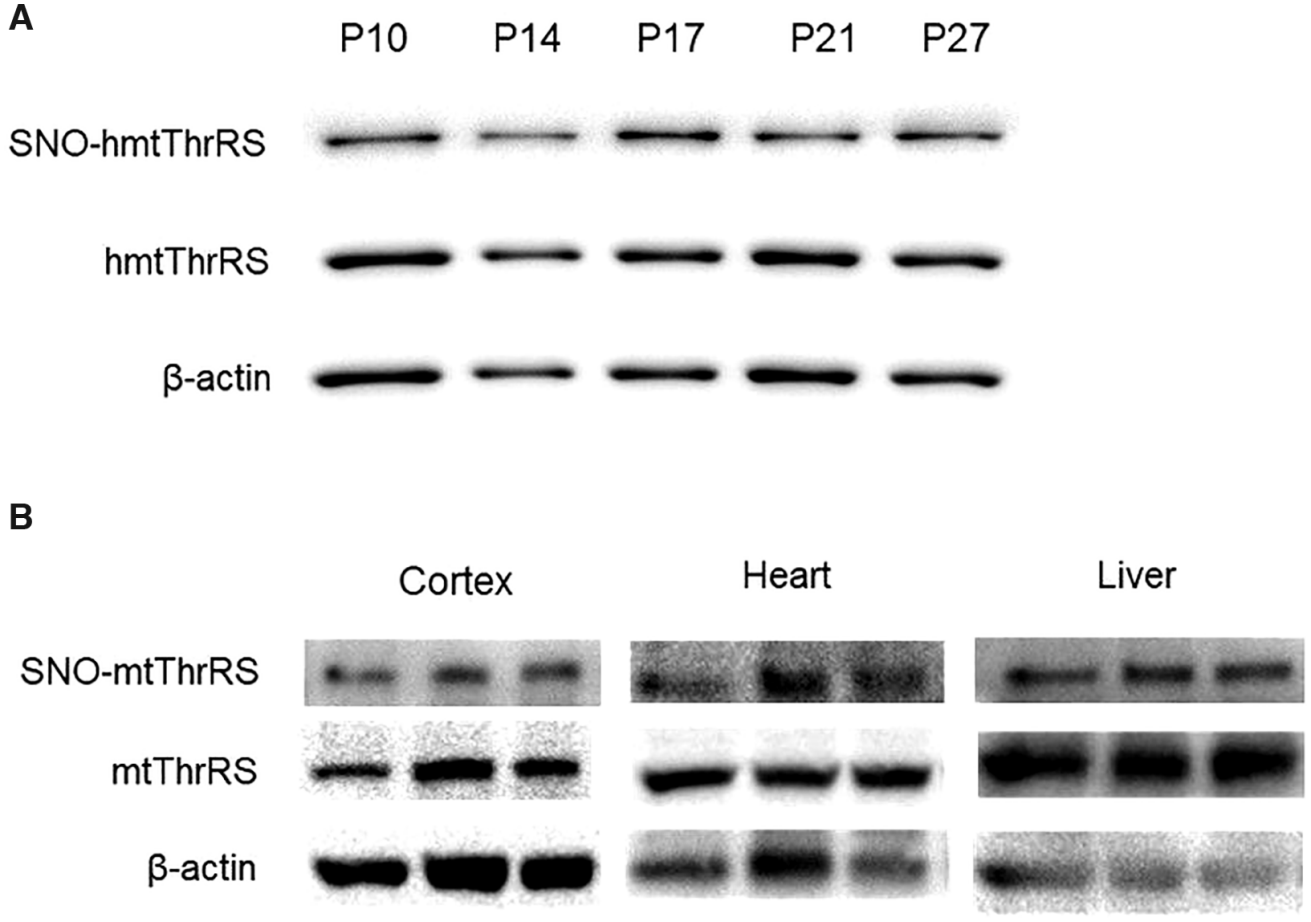
S-nitrosation of mitochondrial ThrRS was readily detected in HDF cell lines and mouse tissues. (**A**) S-nitrosation-modified hmtThrRS was detected in different passages (P10, P14, P17, P21 and P27) of cultured HDF cells. (**B**) S-nitrosation-modified mtThrRS was detected in various mouse tissues, including the cortex, liver and heart.

To explore whether mtThrRS is modified by S-nitrosation *in vivo*, IBP was performed with whole cell lysates of human fibroblast (HDF) cells, and avidin-purified proteins were detected using hmtThrRS antibodies. Indeed, S-nitrosation modification of hmtThrRS in the HDF cells at different stages was readily detected (Figure 5A). Similar procedures were also employed using various tissues of mice, including the cortex, heart, and liver, and S-nitrosation of mtThrRS was also monitored in these tissues (Figure 5B). The results showed that the S-nitrosation modification of mtThrRS is found *in vivo* and may be widespread among human cells and various mouse tissues.

### S-nitrosation-modified human mitochondrial aaRSs

On the basis of the sensitivity of hmtThrRS activities to GSNO-induced S-nitrosation modification *in vitro* (Figure 2, Supplementary Figure S7) and the detection of S-nitrosation-modified hmtThrRS *in vivo* (Figure 5), we also explored whether other mitochondrial aaRSs bear S-nitrosation modification. To this end, human embryonic kidney 293T (HEK293T) cell lysate was incubated with GSNO, and the S-nitrosation-modified mitochondrial aaRSs were determined by IBP-MS (Supplementary Figure 5B). However, no mitochondrial aaRS species were identified, likely because of the low concentration of mitochondrial proteins in the whole cell lysate. Mitochondria were then separated from the HEK293T cells, enriched and then incubated with GSNO (1 mM). The IBP-MS method was then employed to successfully capture 14 Cys residues with S-nitrosation modification on 8 mitochondrial aaRSs: mitochondrial AlaRS, AspRS, GlyRS, GluRS, LeuRS, MetRS, IleRS and ThrRS (Supplementary Table S3). Although 4 Cys residues were identified with GSNO-treated purified hmtThrRS, only one of them, Cys^506^, was captured using GSNO-treated mitochondria, probably because of poorly diffused GSNO and a lower concentration of native hmtThrRS in the matrix of the treated mitochondria. This finding also suggested that Cys^506^ was indeed highly sensitive to GSNO. Analysis based on the crystal structures of various aaRSs revealed that these Cys residues were widely distributed among aminoacylation, editing and the tRNA binding domains. Sequence analysis showed that these Cys residues were mostly nonconserved. These data clearly showed that mitochondrial aaRSs constitute a family of enzymes sensitive to posttranslational S-nitrosation modification mediated by RNS and that we had successfully established a GSNO-sensitive mitochondrial aaRS network.

### S-nitrosation-modified cytosolic aaRSs in mouse cortexes

We readily detected modified hmtThrRS in mouse tissues (cortex, liver and heart) (Figure 5B) and identified several S-nitrosated mitochondrial aaRSs in human HEK293T cells (Supplementary Table S3). Besides being found in mitochondria, RNS was present in abundance in the cytoplasm. To determine whether cytoplasmic aaRSs can also be S-nitrosation-modified, the cortex of adult mice was employed, considering that the cortex is one of the tissues that requires abundant and active protein synthesis for neuronal development, synaptic plasticity, memory acquisition and maintenance. The separated mouse cortex was lysed, and IBP-MS (Supplementary Figure S5B) was performed as described above. A total of 40 modified Cys residues of 11 aaRSs (13 proteins due to two subunits of PheRS and fused GluProRS) were finally established from the mouse cortex *in vivo* (Supplementary Table S4), namely, mouse cytoplasmic AlaRS, AsnRS, AspRS, CysRS, GluProRS, MetRS, PheRS, SerRS, ThrRS, TrpRS and ValRS. Most of the sites of S-nitrosation, similar to the modified sites in mitochondrial aaRSs, were not conserved, except Cys^300^ of the mouse cytoplasmic SerRS, which was highly conserved and located in the aaRS class-defining motif II peptide. Based on the crystal structure of bovine mitochondrial (PDB No. 1WLE) (48) and human cytoplasmic SerRS (PDB No. 4L87) (49), it has no direct interaction with its substrates. In addition, the modified sites were mainly located in the aminoacylation domains (Supplementary Table S4), suggesting that aminoacylation is probably the primarily affected function of S-nitrosation in the cytoplasmic aaRSs. In this procedure, although mitochondrial aaRSs were also contained in the cortex lysate, we identified only one mouse mitochondrial aaRS, mtIleRS, with three S-nitrosated sites, Cys^155^ and Cys^465^ in the aminoacylation domain and Cys^1002^ in its C-terminal tRNA binding domain. Notably, Cys^1002^ was also detected when using GSNO-treated human mitochondria (Supplementary Table S3), suggesting a conserved modification at this site across species.

These data clearly revealed that most of the cytoplasmic aaRSs are indeed modified by S-nitrosation *in vivo* and that we established a cytoplasmic aaRS network with S-nitrosation.

### Alteration in the S-nitrosation-modification of mtThrRS in a huntington mouse model

**Figure 6.**
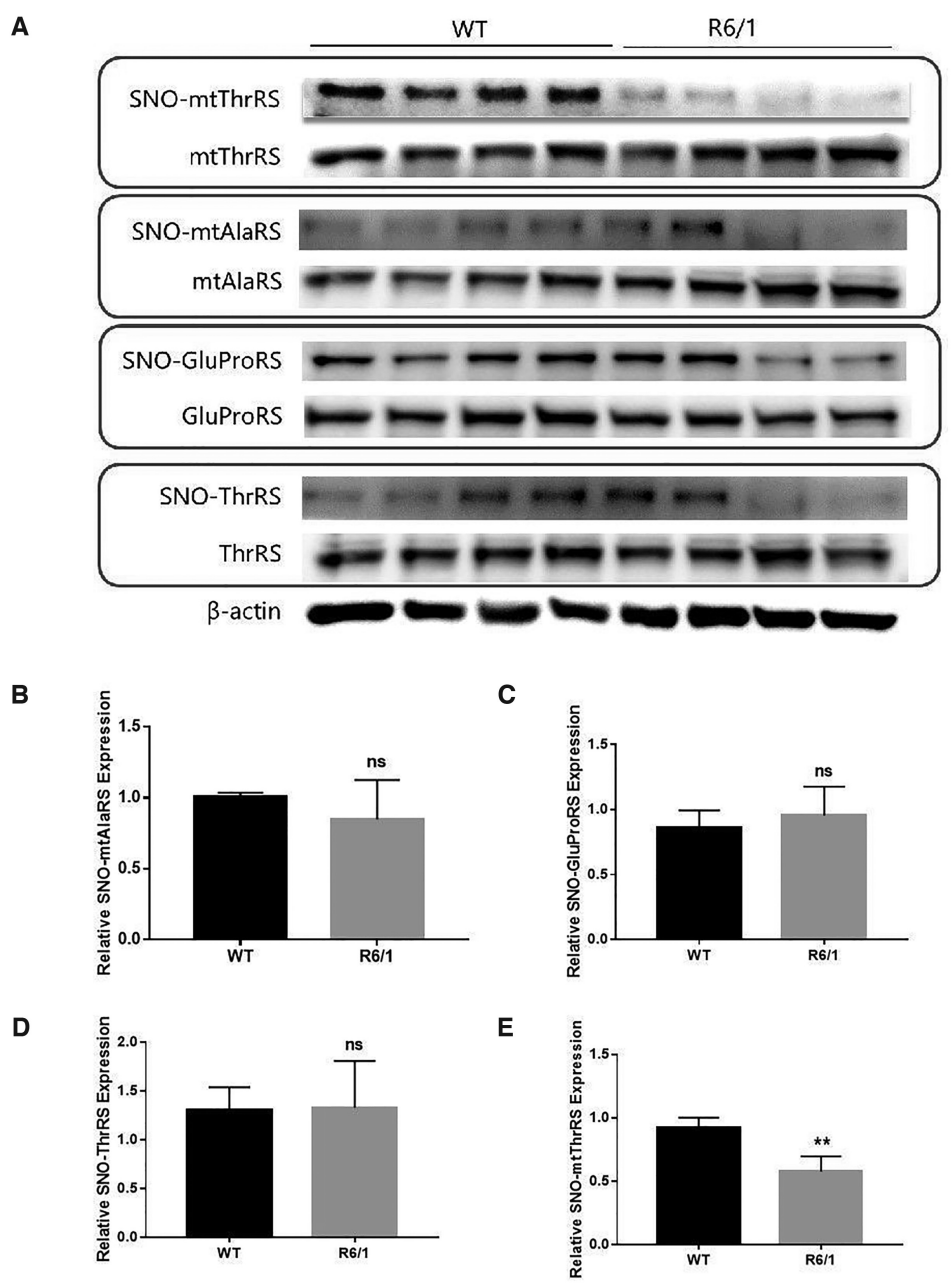
S-nitrosation-modified mtThrRS was decreased in the cortex of Huntington mouse model R6-1 mice. (**A**) S-nitrosation modification of two mitochondrial aaRSs (mtThrRS and mtAlaRS) and two cytoplasmic aaRSs (GluProRS and ThrRS) was detected in the cortex of the R6-1 mice. (B–E) Quantitative analysis of the relative expression levels of S-nitrosation-modified mtAlaRS (**B**), GluProRS (**C**), ThrRS (**D**), and mtThrRS (**E**). All values are reported as the mean ± SD with *n* = 4.

Several independent studies have shown that abnormal protein synthesis is one of the multiple features of diseases associated with cognitive impairment (50–54). Because we have shown the inhibitory role of S-nitrosation in aminoacylation and revealed a bundle of both S-nitrosation-modified cytoplasmic and mitochondrial aaRSs in human cell lines and the mouse cortex, the latter being involved in some cognitive disorders, such as Huntington disease; we used Huntington model R6-1 mice (55) to verify whether modification of some aaRSs was altered. We selected two cytoplasmic aaRSs (ThrRS and GluProRS) and two mitochondrial aaRSs (mtAlaRS and mtThrRS), which have been well identified in *in vivo* detection from mouse cortex. The results clearly showed that the four aaRSs were indeed S-nitrosated *in vivo* in the cortex of wild-type and R6-1 mice. The modification levels of cytoplasmic ThrRS, GluProRS and mitochondrial AlaRS seemed not to be altered (Figure 6A–D). Interestingly, the abundance of modified mouse mitochondrial ThrRS was obviously decreased (Figure 6A and E), suggesting that mitochondrial ThrRS is probably an excellent RNS sensor in the pathogenesis of Huntington disease.

## DISCUSSION

Regulation of the structure and/or function of aaRSs by H_2_O_2_ was reported previously (31,33,34). However, chemical modification and structural/functional regulation of aaRSs by GSNO has never been reported. Summarized in the Supplementary Table S5, it shows the different effects of oxidative/nitrosative stress on the charging or mischarging of tRNA in different species. ThrRS is a typical example of H_2_O_2_-sensitive aaRS (31,32,36). In *E. coli*, H_2_O_2_ reduced global translational fidelity by oxidizing a totally conserved editing active site (Cys^182^) in *Ec*ThrRS, but other sites (Supplementary Table S5) (31), including the synthetic active site Cys^334^ (hmtThrRS Cys^409^ counterpart), were not affected. In contrast, in this work, we found that both hmtThrRS and cytoplasmic ThrRS were insensitive to H_2_O_2_ treatment in terms of aminoacylation and mischarging (Supplementary Table S5). Notably, hmtThrRS also contains a completely conserved editing active site, Cys^240^ (*Ec*ThrRS Cys^182^ counterpart). Conversely, hmtThrRS was obviously sensitive to GSNO treatment *in vitro*. Four Cys residues were found to be modified after exposure to GSNO, one of which was the completely conserved aminoacylation active site, Cys^409^. However, Cys^240^ was not modified by GSNO. These lines of evidence suggest that the same aaRS in different species or organelles has the capacity to respond differently to various redox stresses.

Our results initially showed that S-nitrosation of hmtThrRS decreased both aminoacylation and mischarging activities. Indeed, the four identified modified Cys residues were located at both aminoacylation and editing domains. However, amino acid activation was less affected in comparison with aminoacylation and editing. Considering that Cys^409^ is a crucial residue in Thr binding and activation, the findings implied a lower level of modified Cys^409^ in overall global modification than that of modified Cys residues at other sites. We suggest that modification selectivity of the Cys^409^ site in hmtThrRS was highly significant because this residue has the potential to function as a switch to effectively regulate aminoacylation activity and the corresponding mitochondrial protein synthesis rate in a timely and precise manner, similar to the modification of editing-essential Cys^182^ in *Ec*ThrRS for modulating its proofreading activity and protein mistranslation in *E. coli* under H_2_O_2_ stress (31,32). After mutating all four Cys residues into Ser, the inhibitory effect of GSNO on aminoacylation and editing was completely eliminated. Specifically, despite the impaired editing activity caused by modification, the generated Ser-tRNA^Thr^ was decreased, suggesting that accumulation of Ser-tRNA^Thr^ in the initial mis-aminoacylation step was downregulated as synthesis of Thr-tRNA^Thr^, and was rate-limiting in mischarging. hmtThrRS has 10 Cys residues, prone to be modified by GSNO based on its reactivity with DTNB. The selection of four specific residues for modification might not be random but to realize a common inhibitory effect on enzymatic activities. Indeed, after introducing triple mutations on hmtThrRS, we found that modification at each site was able to decrease aminoacylation and editing activities. We suggest that S-nitrosation of hmtThrRS can function as a new rapid regulatory mechanism in the response to alterations caused by stresses (such as environment, nutrition etc.) and is likely beneficial for mitochondrial function and cell survival. In addition to the impairment of the editing function of *Ec*ThrRS under oxidative stress, increased editing activity was also recently observed with *Se*PheRS after its oxidation by H_2_O_2_. This increase in editing seems to be beneficial for protecting cells from the negative effect of the mistranslation of *meta*-Tyr (m-Tyr), which can rapidly accumulate in cells under oxidative stress (Supplementary Table S5) (33). Furthermore, MetRS, which has no editing function, could be phosphorylated and then mischarges non-tRNA^Met^s to induce adaptive mistranslation, protecting proteins from further oxidation and is thus beneficial to cells (Supplementary Table S5) (34).

Beside redox stress, other physiological or pathological stresses also lead to other types of aaRS modification, regulating enzymatic activity and/or subcellular localization. For example, IFN-γ or viral infection induced phosphorylation of GluProRS (56,57) and antigen-IgE stimulated phosphorylation of LysRS (58) alter their spatial localization and/or canonical aminoacylation activity in noncanonical functions. Acetylation of *E. coli* LeuRS (59) or human TyrRS (60) inhibits aminoacylation activity or promotes nuclear accumulation to prevent cells from DNA damage. Therefore, our results add a new type of modification of aaRSs. All of the above examples, together with S-nitrosation of hmtThrRS, suggest that, different aaRS species have the potential to use multiple modifications and mechanisms to respond to various stresses.

Among the four sites identified with *in vitro*-treated hmtThrRS, only one site (Cys^506^) was successfully captured using purified mitochondria, likely derived from the lower effective GSNO concentration diffusing into mitochondria *ex vivo*. This result also implies that, under profound RNS stress, more sites in more mitochondrial aaRSs might be modified than were identified in this study. In addition, using mouse cortexes, we identified dozens of SNO-modified Cys residues in more than one-half of the cytoplasmic aaRSs. This finding is of particular importance because it directly demonstrates that the S-nitrosation of multiple aaRSs is a naturally occurring regulatory chemical modification *in vivo* under physiological conditions. It also suggests that RNS-mediated aaRS modification is a more comprehensive mechanism for profoundly regulating aaRS structure and/or function, which has been previously overlooked. Overall, our work, for the first time, established both cytoplasmic and mitochondrial aaRS regulatory networks with S-nitrosation modification. We anticipate that more sites and more aaRS species with S-nitrosation will be discovered under various RNS stresses with advances in the sensitivity of detection and quantification methods.

The distribution of the modified sites in various domains suggested multiple and profound regulatory effects of both cytoplasmic and mitochondrial aaRSs. These effects may include changed affinity of the enzymes for amino acids, ATP or tRNA and subsequent alterations in the activities of aminoacylation and/or editing. These possibilities are likely most relevant for SerRS because one of its aminoacylation active sites, Cys^300^, is readily detected as SNO-modified. Cys^300^ is conserved in most species and is located in a crucial helix in the ATP- and Ser-binding pocket in the synthetic site, as indicated by the cytoplasmic (49) and mitochondrial SerRS structures (48). It is very likely that modification of this site in class II-defining peptide introduces an additional SNO group in the substrate-binding pockets and thus significantly inhibits SerRS synthetic activity. The second effect may be involved in alterations to protein-protein interactions in the canonical or noncanonical functions of aaRSs. This effect is especially relevant for GluProRS, for example, because it harbors three modified Cys residues (Cys^680^, Cys^692^ and Cys^697^) in the WHEP domain, which is critical for the incorporation of GluProRS into the multiple tRNA synthetase complex (MSC), composed of nine aaRSs and three non-aaRS co-factors (61). In addition, ValRS (also identified in this study) forms a complex with four subunits of elongation factor EF-1H. It is possible that SNO modification disassociates the subunits in these complexes or otherwise affects protein-protein interactions. Indeed, phosphorylation at Ser^886^ and Ser^999^ or at Ser^990^ in the WHEP domain efficiently released GluProRS from the MSC to perform noncanonical functions (56,57). Notably, S-nitrosation is found in six aaRS components in the MSC. In addition, S-nitrosation of aaRSs may also have the potential to regulate its structure (due to local conformational changes), steady-state level in the cells (due to influencing solubility), mitochondrial import efficiency (for mitochondrial aaRSs) *etc*., because Cys modification possibly influences intramolecular and/or intermolecular interactions by disrupting the formation of disulfide bonds.

As an important functional mechanism of nitric oxide *in vivo*, accumulating reports have indicated that S-nitrosation modification of a protein plays a key role in normal physiology and many human diseases (24,62). A series of abnormal S-nitrosated modified protein targets, including Drp1, Cdk5, PDI and Parkin, were found in neurodegenerative diseases. S-nitrosation of these protein targets is involved in mitochondrial fission, synaptic dysfunction, protein misfolding and ER stress (25). Our previous work has also revealed the important role of S-nitrosated modified protein targets, including ATG4B, HDAC2 and CaMKIIα, in neurotoxicity, neuronal differentiation and age-related cognitive impairment (28,63,64). S-nitrosation of mitochondrial ThrRS has been shown to transpire naturally *in vivo* in multiple mouse tissues, including the cortex, liver and heart. To our knowledge, mtThrRS is the first identified protein target to undergo S-nitrosylation modification in aaRSs. Abnormally increased levels of protein synthesis have been reported in many neurodegenerative diseases, including Huntington disease. The literature has also reported exaggerated translation in Huntington's disease model R6-1 mice, and pharmacological normalization of protein synthesis by translation inhibitors ameliorates motor disturbances in R6-1 mice (50). In this work, we first proved that S-nitrosation of mtThrRS was significantly downregulated in the cortex of Huntington's model R6-1 mice, implying an outcome potentially associated with an increase in protein synthesis. However, the direct relationship needs to be further explored. We proposed that mtThrRS is a newly discovered abnormal S-nitrosated modified protein target in neurodegenerative diseases and is likely associated with the progression of neurodegenerative diseases by regulating protein synthesis. Certainly, there are still some unsolved issues in the possible linkage of mtThrRS modification alteration and Huntington diseases, including the reason, effect, and mechanism of decreased modification, which are worthy of studying in the future.

## Supplementary Material

gkaa471_Supplemental_File
