# Supplementary material for: Nitrosative stress inhibits aminoacylation and editing activities of mitochondrial threonyl-tRNA synthetase by S-nitrosation
Source: Nucleic Acids Res. 2020 Jun 2;48(12):6799–810. doi: 10.1093/nar/gkaa471 (PMC7337905; doi:10.1093/nar/gkaa471)
Supplement: gkaa471_Supplemental_File [file gkaa471_supplemental_file.docx]

**
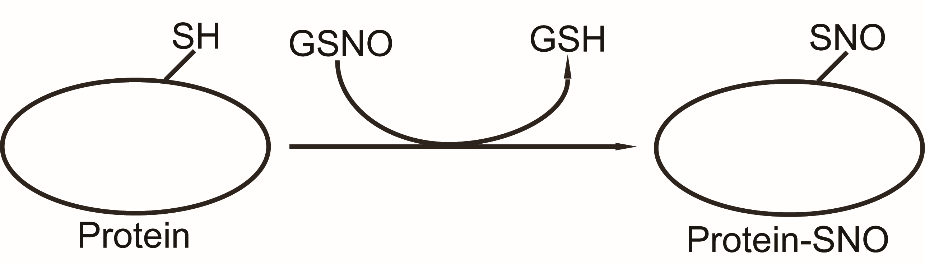
**

**Supplementary Figure 1. A schema showing protein S-nitrosation mediated by GSNO *in vitro*.**

**

**

**Supplementary Figure 2**. Effect of GSNO treatment on the amino acid activation activity of hmtThrRS. The ATP-PPi exchange reaction was performed with hmtThrRS without (red) or with (blue) GSNO treatment.

**
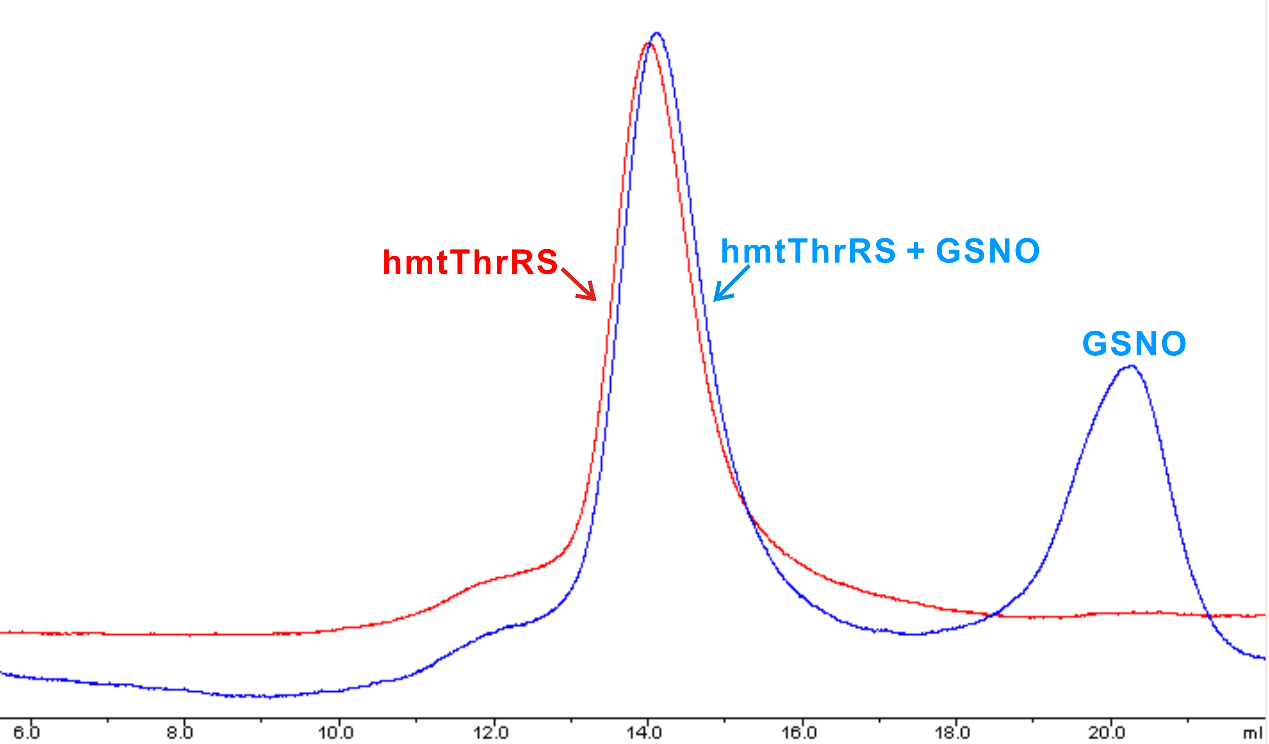
**

**Supplementary Figure 3**. hmtThrRS dimerization is not influenced by GSNO treatment. 0.4 mg/ml (5 μM) hmtThrRS was incubated without (red) or with (blue) 5 mM GSNO in 500 μl of buffer containing 60 mM Tris-HCl (pH 7.5) and 10 mM MgCl_2_, and then, subjected to gel filtration analysis (Superdex 200 increase column), showing that hmtThrRS remains a dimer after GSNO treatment.


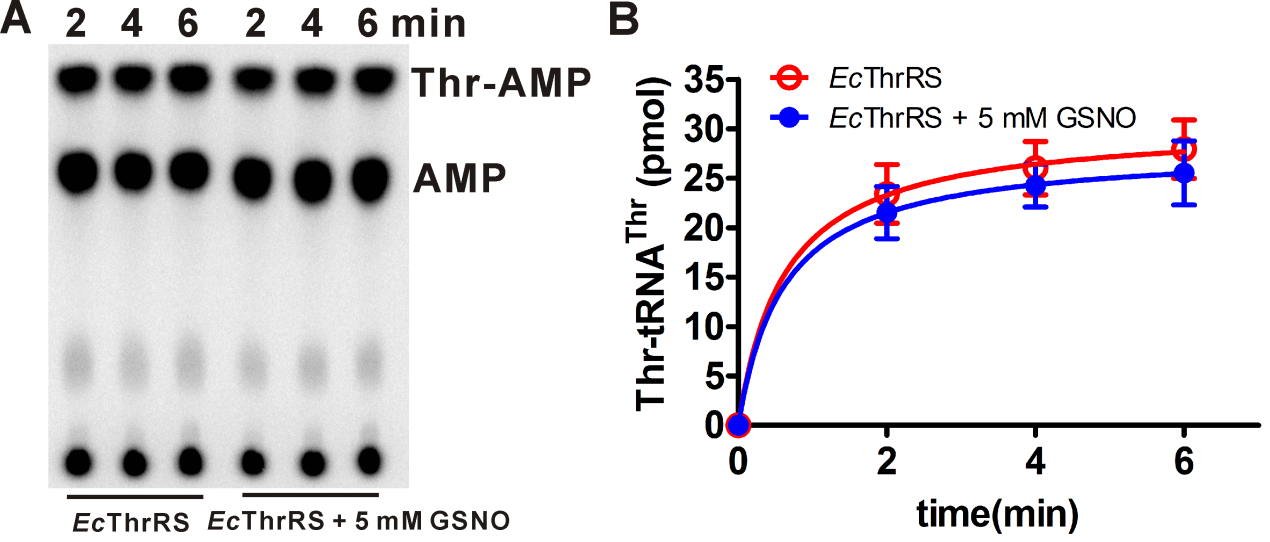


**Supplementary Figure 4**. The aminoacylation of *Ec*ThrRS is not regulated by GSNO. (A) A representative graph showing aminoacylation of [^32^P]tRNA^Thr^ with Thr by *Ec*ThrRS in the absence or presence of 5 mM GSNO treatment as indicated. (B) Amount of Thr-[^32^P]tRNA^Thr^ catalyzed by *Ec*ThrRS without (red) or with (blue) GSNO treatment, as in (A).


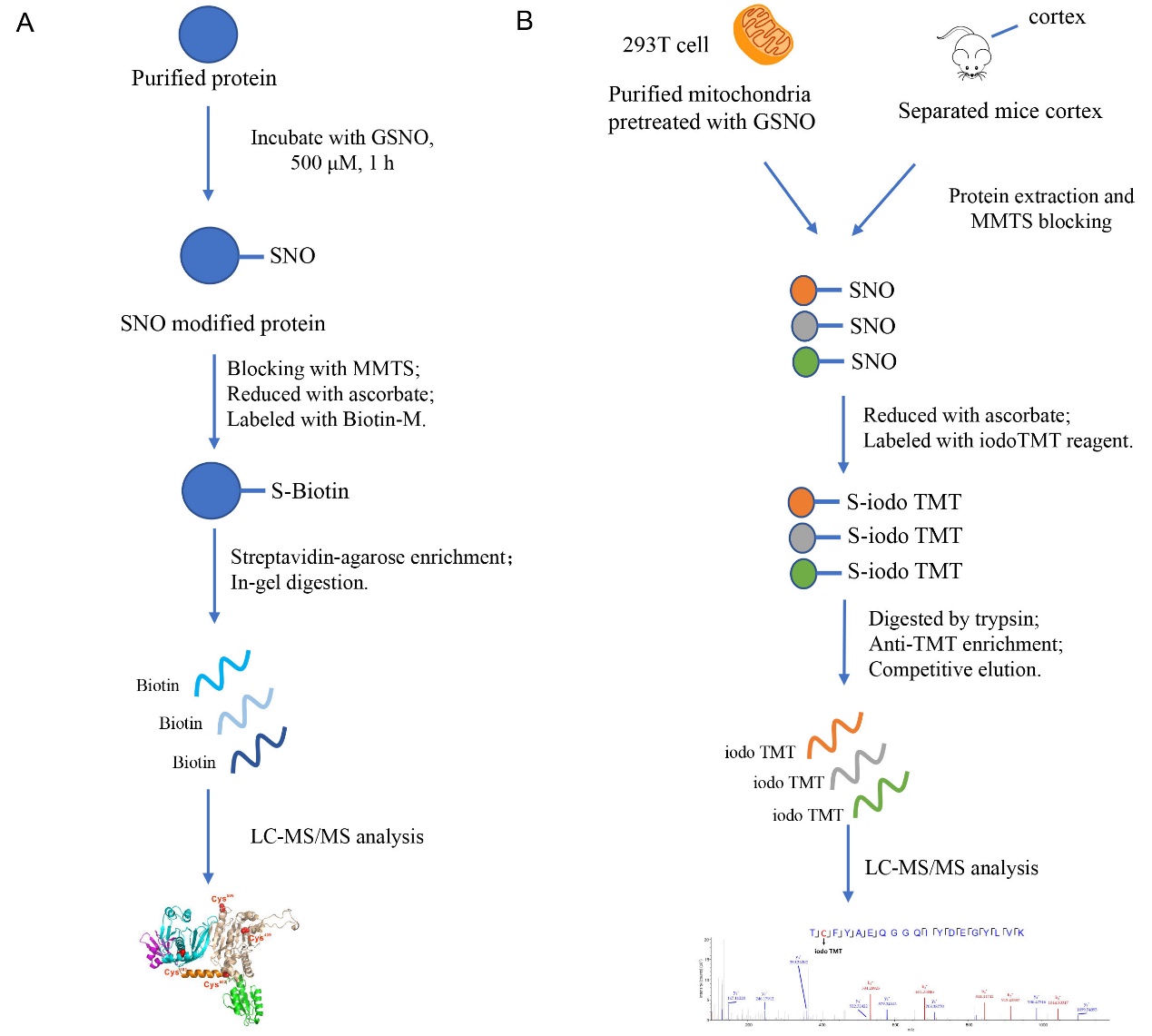


**Supplementary Figure 5**. Schematic overview of the IBP-MS method. (A) Purified protein pretreated with GSNO (500 μM for 1 h) and S-nitrosated Cys residues were labeled with biotin-M reagent and enriched with streptavidin-agarose. (B) Purified human mitochondria pretreated with GSNO (1 mM for 1 h) or separated mouse cortex were lysed in HENS buffer, free Cys residues were blocked with MMTS, and S-nitrosated Cys residues from different protein targets were labeled with iodoTMT reagent and enriched with anti-TMT antibody. Then, the digested peptides were analyzed by MS detection to find biotin (A)/iodoTMT (B)-modified protein targets and the Cys sites.





**Supplementary Figure 6**. Primary sequence analysis of peptides covering four modified Cys residues in ThrRSs and cytoplasmic ThrRS-like proteins (ThrRS-L, encoded by *TARS3*). Conserved and homologous residues are highlighted in black and gray, respectively. hmt, human mitochondrial; mmt, mouse mitochondrial; *Bt*, *Bos taurus*; *Rn*, *Rattus norvegicus*; hThrRS(-L), human cytoplasmic ThrRS(-L); mThrRS(-L), mouse cytoplasmic ThrRS(-L); *Tc*, *Trypanosoma cruzi*; *Sc*, *Saccharomyces cerevisiae*; *Ca*, *Candida albicans*; *Ec*, *Escherichia coli*; *Hp*, *Helicobacter pylori*; and *Bs*, *Bacillus subtilis*.

**
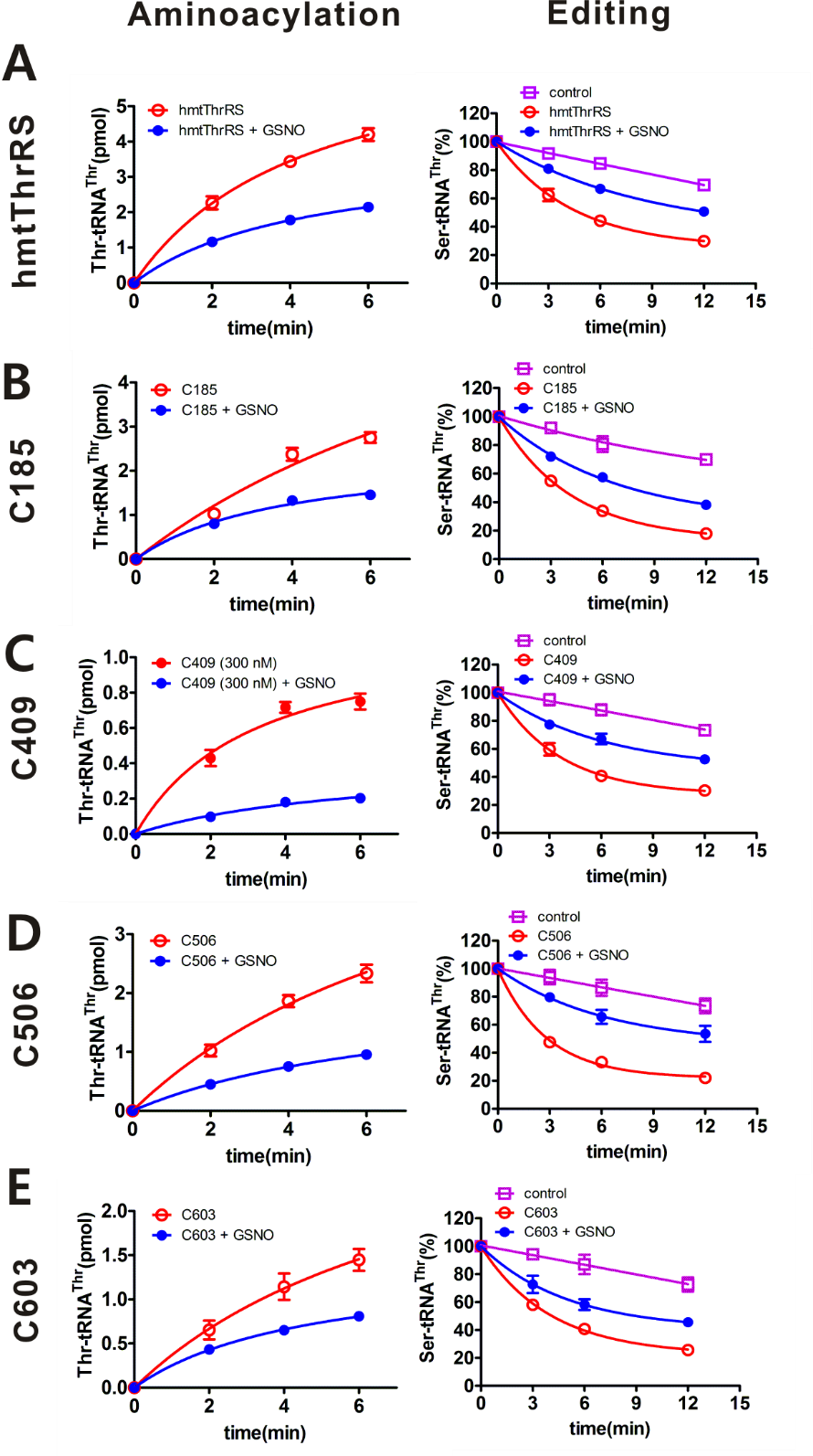
**

**Supplementary Figure 7**. Inhibition of aminoacylation and editing activities of various mutants by GSNO. Aminoacylation of [^32^P]tRNA^Thr^ (left) or posttransfer editing of pre-formed Ser-[^32^P]tRNA^Thr^ (right) was determined with GSNO untreated (red) or treated (blue) hmtThrRS (A), C185 (B), C409 (C), C506 (D), and C603 (E). A final concentration of 300 nM of the C409 mutant was used for aminoacylation determination due to its low activity. In all posttransfer editing assays, a control reaction (purple) represents the spontaneous hydrolysis of Ser-[^32^P]tRNA^Thr^ without the addition of enzyme. Reaction conditions were described in detail in the “MATERIALS AND METHODS”.

**Tables**

| **Supplementary Table 1. Number of Cys residues with DTNB reactivity** | |
| --- | --- |
| Protein | Number of Cys residues |
| Native hmtThrRS | 10.0 ± 0.03 |
| hmtThrRS + GdnHCl | 13.1 ± 0.03 |
| Values are reported as the mean ± SD of three independent determinations. | |

| **Supplementary Table 2. *Ex vivo* detection of purified hmtThrRS with S-nitrosation modification** | | | |
| --- | --- | --- | --- |
| Site | Sequence | | M/Z [Da] |
| Cys^185^ | | IC*QELTAAAR | 763.8774 |
| Cys^409^ | | HITDTLALKPMNC*PAHcLMFAHRPR | 677.1309 |
| Cys^506^ | | LALSTRPSGFLGDPC*LWDQAEQVLK | 1065.872 |
| Cys^603^ | | LLGVLAESC*GGK | 799.4072 |

| **Supplementary Table 3. *Ex vivo* detection of human mitochondrial aaRSs with S-nitrosation modification** | | | | |  |
| --- | --- | --- | --- | --- | --- |
| **Enzyme** | **Sequence (modified sites, bold red)** | **Positions** | **Domain** | | |
| mtAlaRS | PSGSYEFGT**C**EAQVLQLYTEDGTAVASVGK | Cys^531^ | | Editing |  |
|  | HSTYDTDLFSPLLNAIQQGCR | Cys^300^ | | Aminoacylation |  |
|  | EEA**C**NMAWELLTQVYGIPEER | Cys^149^ | | Aminoacylation |  |
|  | AQV**C**GGFILHEAVAPE**C**LR | Cys^596^, Cys^609^ | | Editing |  |
|  |  |  | |  |  |
| mtAspRS | TNTCGELR | Cys^54^ | | aminoacylation |  |
|  | TAELLNACK | Cys^449^ | | Insertion |  |
|  |  |  | |  |  |
| mtGlyRS^a^ | SCYDLSCHAR | Cys^466^ | | Ins3 domain |  |
|  |  |  | |  |  |
| mtGluRS | TGAAYPCFCSPQR | Cys^140^ | | Aminoacylation |  |
|  |  |  | |  |  |
| mtLeuRS | EALVNWDPVDQTVLANEQVDEHGCSWR | Cys^225^ | | Aminoacylation |  |
|  |  |  | |  |  |
| mtMetRS | FYGHPCPFEGR | Cys^562^ | | tRNA binding |  |
|  |  |  | |  |  |
| mtIleRS | RPYWCISR | Cys^521^ | | Aminoacylation |  |
|  | YTAESSDTLCPR | Cys^1002^ | | tRNA binding |  |
|  |  |  | |  |  |
| mtThrRS | PSGFLGDPCLWDQAEQVLK | Cys^506^ | | Aminoacylation |  |
| ^a,^ GlyRS is a dually localized aaRS. | | | | |  |

| **Supplementary Table 4. *In vivo* detection of mouse cytoplasmic aaRSs with S-nitrosation modification** | | | |
| --- | --- | --- | --- |
| **Enzyme** | **Sequence (modified sites, bold red)** | **Positions** | **Domain** |
| AlaRS | DNFWEMGDTGP**C**GP**C**SEIHYDR | Cys^184^, Cys^187^ | Aminoacylation |
|  | IQSLGD**C**K | Cys^403^ | tRNA acceptor binding |
|  | T**C**FYAEQGGQIYDEGYLVK | Cys^533^ | Editing |
|  | MFVDEVVTGQE**C**GVVLDK | Cys^525^ | Editing |
|  | IT**C**L**C**QVPQNAANR | Cys^901^, Cys^903^ | C-terminal helical domain |
|  |  |  |  |
|  |  |  |  |
| AsnRS | NDPSLPEPA**C**VK | Cys^114^ | Anticodon-binding domain |
|  | GY**C**EVTTPTLVQTQVEGGATLFK | Cys^266^ | Aminoacylation |
|  | LMTDTINEPILL**C**R | Cys^438^ | Aminoacylation |
|  | DV**C**LYPR | Cys^537^ | Aminoacylation |
|  |  |  |  |
| AspRS | VF**C**IGPVFR | Cys^267^ | Aminoacylation |
|  | LEY**C**EALAMLR | Cys^349^ | Aminoacylation |
|  |  |  |  |
| CysRS | VQPQWSPPAGTEP**C**R | Cys^110^ | Aminoacylation |
|  | FPHHDNELAQSEAYFEND**C**WVR | Cys^389^ | Aminoacylation |
|  |  |  |  |
| GluProRS | HEELMLGDP**C**LK | Cys^660^ | Anticodon-binding domain |
|  | GFFI**C**DQPYEPVSPYS**C**R | Cys^680^, Cys^692^ | WHEP domain |
|  | EAP**C**ILIYIPDGHTK | Cys^697^ | WHEP domain |
|  |  |  |  |
| MetRS | FFGG**C**VPEMALTPDDR | Cys^670^ | tRNA binding domain |
|  |  |  |  |
| PheRS-α | **C**WELTTEGEEIAR | Cys^60^ | tRNA binding domain |
|  | VNLQMVYDSPV**C**R | Cys^493^ | Aminoacylation |
|  |  |  |  |
| PheRS-β | YDLL**C**LEGLAR | Cys^76^ | B3 domain |
|  | EYTA**C**ELMNIYK | Cys^195^ | B4 domain |
|  |  |  |  |
| SerRS | IWGD**C**TVR | Cys^162^ | Aminoacylation |
|  | YAGLST**C**FR | Cys^300^ | Aminoacylation |
|  | ELVS**C**SN**C**TDYQAR | Cys^395^, Cys^398^ | Aminoacylation |
|  | TI**C**AILENYQAEK | Cys^438^ | Aminoacylation |
|  |  |  |  |
| ThrRS | DQELYFFHELSPGS**C**FFLPK | Cys^342^ | Aminoacylation |
|  |  |  |  |
| TrpRS | DIIA**C**GFDINK | Cys^229^ | Aminoacylation |
|  | GIFGFTDSD**C**IGK | Cys^278^ | Aminoacylation |
|  |  |  |  |
| ValRS | DNPMVVPL**C**NR | Cys^662^ | Editing |
|  | ADFPAGIPE**C**GTDALR | Cys^916^ | Aminoacylation |
|  | APASL**C**VTPYPEPSECSWK | Cys^1086^, Cys^1096^ | Aminoacylation |

| **Supplementary Table 5. Summary of different effects of oxidative/nitrosative stress on charging or mischarging of tRNAs in different species** | | | | | |
| --- | --- | --- | --- | --- | --- |
| aaRS | Charging | | Mischarging | ROS/RNS | Refs. |
| *Ec*ThrRS | | ND | ↑ | H_2_O_2_ | Refs. 31, 32 |
| *Se*PheRS | | --- | ↓ |  | Ref. 33 |
| human, yeast cytoplasmic or bacterial MetRS | | ND | ↑ |  | Refs. 34, 35 |
| mThrRS | | --- | --- |  | this work |
| hmtThrRS | | --- | --- |  | this work |
| hmtThrRS | | ↓ | ↓ | GSNO | this work |
| ---, not affected; ND, not determined. | | | | | |
